# HDL-C/apoA-I Ratio Is Associated with the Severity of Coronary Artery Stenosis in Diabetic Patients with Acute Coronary Syndrome

**DOI:** 10.1155/2021/6689056

**Published:** 2021-05-18

**Authors:** Lizhe Sun, Manyun Guo, Chenbo Xu, Xiangrui Qiao, Yiming Hua, Gulinigaer Tuerhongjiang, Bowen Lou, Ruifeng Li, Xiaofang Bai, Juan Zhou, Yue Wu, Jianqing She, Zuyi Yuan

**Affiliations:** ^1^Department of Cardiovascular Medicine, The First Affiliated Hospital, Xi'an Jiaotong University, Xi'an, Shaanxi, China; ^2^Department of Ultrasound Imaging, First Affiliated Hospital of Medical College, Xi'an Jiaotong University, Xi'an, Shaanxi, China; ^3^Key Laboratory of Environment and Genes Related to Diseases, Xi'an Jiaotong University, Ministry of Education, Xi'an, Shaanxi, China; ^4^Key Laboratory of Molecular Cardiology of Shaanxi Province, Xi'an, Shaanxi, China

## Abstract

**Background:**

Emerging evidence demonstrates that the lipid metabolism in acute coronary syndrome (ACS) patients with type 2 diabetes mellitus (T2DM) differs from nondiabetic patients. However, the distinct lipid profiles and their relationships with the severity of coronary artery stenosis and prognosis in patients with T2DM remain elusive.

**Method and Result:**

This single-center, prospective cohort study enrolled 468 patients diagnosed with ACS undergoing coronary angiography, consisting of 314 non-DM and 154 DM patients. The HDL-C/apoA-I ratio was significantly higher in DM patients with a multivessel (≥3 affected vessels) lesion than a single-vessel (1-2 affected vessels) lesion. Regression analyses showed that the HDL-C/apoA-I ratio was positively correlated to the number of stenotic coronary arteries in DM patients but not non-DM patients. However, Kaplan-Meier survival analysis revealed no significant difference in the major adverse cardiovascular event rate regarding different HDL-C/apoA-I levels in DM or non-DM ACS patients at the end of the 2-year follow-up.

**Conclusion:**

A higher HDL-C/apoA-I ratio is associated with increased severity of coronary artery stenosis in DM patients with ACS but not with the rate of major adverse cardiovascular events at the end of the 2-year follow-up.

## 1. Introduction

Acute coronary syndrome (ACS) is one of the major public health problems worldwide, which describes the range of acute myocardial ischemic states, including unstable angina (UA), non-ST elevated myocardial infarction (NSTEMI), and ST elevated myocardial infarction (STEMI) [1]. Hyperglycemia and dyslipidemia are risk factors for ACS [2]. Numerous studies have revealed that type 2 diabetes mellitus (T2DM) patients with ACS suffer from worse outcomes compared to their nondiabetic peers [3–6]. Our previous study demonstrated that hemoglobin A1c (HbA1c) was positively correlated with the severity of coronary artery stenosis in both diabetic and nondiabetic patients with ACS [7]; however, the underlying pathophysiological mechanism and clinical manifestations remain to be elucidated.

High-density lipoprotein cholesteryl (HDL-C) was ascribed as “good” cholesteryl and negatively correlated to the risk of cardiovascular diseases, as proven by several clinical and animal studies [8, 9]. The mechanisms of the antiatherogenic effects of HDL-C have been proved to be related to its involvement in the pathways of reverse cholesteryl transport, as well as antioxidation, anti-inflammation, and endothelial protection [8, 10]. However, HDL from T2DM patients showed impaired endothelial-protective capacities due to reduced endothelial progenitor cell-mediated endothelial repair [11]. A systemic review of 14 studies has also revealed that the anti-inflammatory effect of HDL was diminished in individuals with T2DM, although the underlying mechanisms remain to be elucidated [12]. These indicate that HDL may undergo functional remodeling during the progression of chronic inflammatory and metabolic diseases.

Apolipoprotein A-I (apoA-I) is the primary functional apolipoprotein component of HDL participating in cholesteryl traffic via multiple mechanisms [13]. For instance, apoA-I plays pivotal roles in the reverse cholesteryl transport pathway by modulating HDL-C formation and stabilization, binding to hepatic scavenger receptors, and activating lecithin-cholesteryl acyltransferase [14]. Overexpression of the human apoA-I gene on apoE^−/−^ and LDLr^−/−^ mice provided long-term protection on diet-induced atherosclerosis [15, 16]. A previous study indicated that the HDL-C/apoA-I ratio is a more effective marker for coronary artery disease than HDL-C alone [17]. However, it is unknown whether the HDL-C/apoA-I ratio correlates to the severity of coronary artery stenosis and prognosis of diabetic ACS patients.

In this present single-center, prospective study, by analyzing the circulating lipid profile and the major adverse cardiovascular events (MACEs) among ACS patients with or without DM, we aimed to evaluate the relationship between the HDL-C/apoA-I ratio and the severity of coronary artery stenosis and also to explore the short-term prognostic value of the HDL-C/apoA-I ratio in ACS patients.

## 2. Research Design and Methods

### 2.1. Study Design and Participants

This is a single-center, prospective cohort study in which 468 ACS patients were recruited, consisting of 229 UA, 71 NSTEMI, and 168 STEMI patients consecutively admitted to the First Affiliated Hospital of Xi'an Jiaotong University from January to December 2016. This study excluded patients who had severe noncardiac disease with an expected survival of less than 1 year, severe renal disease (plasma creatinine ≥ 130 *μ*mol/L), and chronic liver disease (alanine aminotransferase (ALT) ≥ 2 times the upper limit of normal) or over the age of 80 years. Written informed consent was obtained from all study participants with ethical committee approval from the First Affiliated Hospital of Xi'an Jiaotong University.

### 2.2. Data Collection and Laboratory Measurement

Baseline characteristics and clinical data were recorded from patients' standard medical records. Blood HbA1c levels of all patients were measured within 3 h of admission using a Siemens DCA analyzer for quantitative assay. Both the concentrations of specific HbA1c and total hemoglobin were measured. The ratio was reported as percent HbA1c.

Venous blood samples were collected in the morning following an overnight fast for other baseline laboratory measurements. TC was detected using a detection kit from FUJIFILM™ via the HMMPS method; HDL-C, LDL-C, and VLDL-C were detected using a detection kit via the direct measurement method from FUJIFILM™; apoA, apoB, and apoE were measured using a detection kit from SEKISUI™ by turbidimetric inhibition immunoassay. All laboratory assays were performed in duplicate, and the results were averaged.

### 2.3. Assessment of Coronary Artery Stenosis

Selective coronary angiography was performed in multiple views by experienced clinicians. The severity of ACS was characterized by the number of coronary vessels with stenosis (>50% of the lumen diameter). A single-vessel lesion was defined as 1-2 affected vessels, and a multivessel lesion was defined as at least 3 affected vessels.

### 2.4. Outcome and Follow-Up

Follow-up information was obtained via telephone questionnaires or interviews in the hospital by the general practitioner. All-cause death, heart failure, nonfatal MI, and symptom-driven revascularization were defined as MACEs.

### 2.5. Statistical Analysis

All statistical analyses were performed using SPSS 18.0. Data were presented as frequencies and percentages for categorical variables and mean ± SD for continuous variables unless otherwise indicated. Differences between two independent groups were compared using the chi-squared test for categorical data, *t*-test for normally distributed data, and nonparametric test for nonnormally distributed data. One-way ANOVA was used to compare continuous variables among multiple groups. Univariate linear regression analysis was used for calculating the correlation between the HbA1c, the HDL-C/apoA-I ratio, and the severity of coronary artery stenosis. Multivariate regression analysis was conducted to assess the independent contribution of different factors to coronary artery stenosis. DM and non-DM patients were divided into three groups based on tertiles of the HDL-C/apoA-I level. Kaplan-Meier survival curve analysis was conducted to represent the proportional risk of MACE for the HDL-C/apoA-I ratio in patients with or without DM. All probability values were two-tailed. *p* < 0.05 was considered statistically significant.

## 3. Results

### 3.1. Study Population and Baseline Characteristics

**Figure 1:**
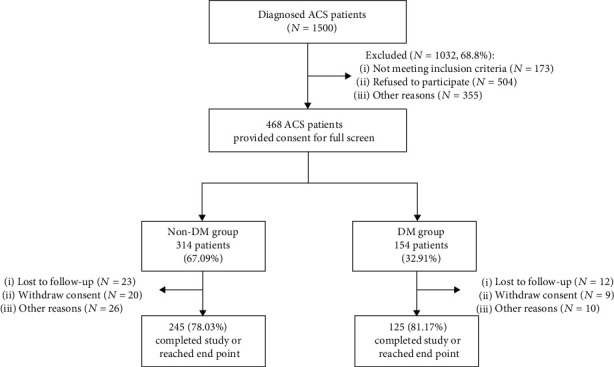
Study flowchart.

A total of 1500 patients with a diagnosis of ACS were screened, of which 173 patients did not meet the inclusion criteria, 504 refused to participate, and 355 were not included in this study due to other reasons. 468 ACS patients were enrolled in the observational study, consisting of 314 non-DM (67.09%) and 154 DM (32.91%) patients. At the end of this study, 245 of 314 non-DM patients (78.03%) and 125 of 154 DM patients (81.71%) completed the 2-year follow-up survival analysis or reached endpoints (Figure 1).

Baseline characteristics of all patients and patients in the non-DM/DM subgroups are shown in Table 1. The 468 ACS patients had a mean age of 60.97 ± 9.57 years with a mean HbA1c of 6.4% ± 1.31 and a mean HDL-C/apoA-I ratio of 0.85 ± 0.12 mmol/g. The mean number of affected vessels indicated by coronary angiography was 2.57 ± 0.87 in DM patients, significantly higher than that in non-DM patients (2.39 ± 0.89, *p* < 0.05). Additionally, the HbA1c level was also higher in DM patients compared to non-DM ACS patients (7.75 ± 1.50 vs. 5.74 ± 0.35, *p* < 0.001). Although no differences were found in low-density lipoprotein cholesteryl (LDL-C), HDL-C, and apoA-I, the HDL-C/apoA-I ratio was lower in diabetic ACS patients than in non-DM patients (0.82 ± 0.10 vs. 0.86 ± 0.12, *p* < 0.01). No differences in other risk factors were found between the non-DM and DM ACS patients, such as age, gender, family history of coronary artery disease, history of hypertension, heart rate (HR), creatine, blood urea nitrogen (BUN), and medication at discharge.

### 3.2. Baseline Characteristics of Patients with Single- and Multivessel Lesions

Baseline characteristics of ACS patients with single-vessel (1-2 affected vessels) and multivessel (≥3 affected vessels) lesions are shown in Table 2. No differences were found in risk factors, including age, gender, BMI, heart rate, systolic/diastolic blood pressure (BP), creatine, left ventricular ejection fraction (LVEF), HDL-C, and LDL-C, between the single- and multivessel lesion groups in either all, non-DM, or DM ACS patients.

In the whole population, 213 patients had multivessel lesions, while 255 patients had a single-vessel lesion. HbA1c was higher in patients with multivessel lesions (6.57 ± 1.45 vs. 6.24 ± 1.14, *p* < 0.05). However, apoA-I is lower in patients with multivessel lesions than a single-vessel lesion (1.05 ± 0.18 vs. 1.09 ± 0.17, *p* < 0.05).

In the non-DM subgroup, 165 and 149 patients had multi- and single-vessel lesions, respectively. HbA1c was unaltered between patients with different numbers of affected vessels, whereas apoA-I was decreased in patients with multivessel lesions compared to those with a single-vessel lesion (1.05 ± 0.18 vs. 1.1 ± 0.17, *p* < 0.05).

In the DM subgroup, 90 and 64 patients had multi- and single-vessel lesions, respectively. HbA1c was 8.03 ± 1.54 in patients with multiple-vessel lesions, which is significantly higher than that in patients with a single-vessel lesion (7.41 ± 1.38, *p* < 0.05). Moreover, the HDL-C/apoA-I ratio was higher in multivessel lesion DM patients than in single-vessel lesion DM patients (0.84 ± 0.11 vs. 0.79 ± 0.08, *p* < 0.05).

### 3.3. Association between HDL-C/apoA-I and Severity of Coronary Artery Stenosis

**Figure 2:**
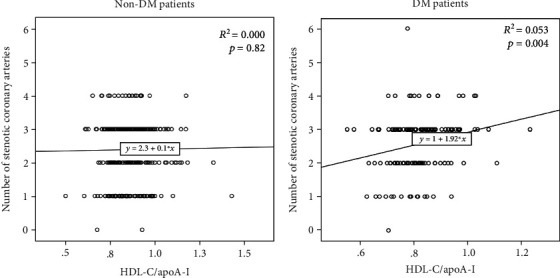
Linear regression analysis between the HDL-C/apoA-I ratio and the number of stenotic coronary arteries in non-DM and DM ACS patients.

The severity of coronary artery stenosis was evaluated by the number of affected vessels suffering from coronary artery stenosis as described. Simple linear regression analysis demonstrated that the HDL-C/apoA-I ratio was positively correlated with the severity of coronary artery stenosis in DM ACS patients (*R*^2^ = 0.053, *p* = 0.004). However, the HDL-C/apoA-I ratio was not associated with the severity of coronary artery stenosis in non-DM ACS patients (Figure 2).

Multiregression analysis was then performed to further determine the risk factors regarding the severity of coronary artery stenosis (Table 3). Consistently, the HDL-C/apoA-I ratio was only found to be significantly positively correlated to the severity of coronary artery stenosis in diabetic ACS patients (95% CI 0.235 to 3.368, *p* < 0.05).

### 3.4. Comparison of Characteristics between Patients with Different HDL-C/apoA-I Levels

Patients were divided into 3 groups based on HDL-C/apoA-I tertiles, and the comparison of various characteristics is shown in Table 4. In the whole population, age, HDL-C, LDL-C, and apoA-I were significantly increased in uprising HDL-C/apoA-I levels (*p* < 0.05). On the contrary, BMI was stepwise decreased based on HDL-C/apoA-I levels (*p* < 0.001). Patients with the middle level of HDL-C/apoA-I had the highest level of HbA1c compared to those with low and high levels of HDL-C/apoA-I (6.52 ± 1.52 vs. 6.45 ± 1.27 vs. 6.22 ± 1.06, *p* < 0.01). No difference in gender, HR, BP, LVEF, and the number of stenotic coronary arteries was found in this comparison. Similar trends of all these characteristics were found in the non-DM ACS patients.

In the DM ACS patient group, age and HDL-C were also increased as HDL-C/apoA-I levels rise (58.92 ± 8.27 vs. 63.1 ± 9.05 vs. 64.22 ± 8.79, *p* < 0.05; 0.76 ± 0.11 vs. 0.9 ± 0.17 vs. 1.08 ± 0.24, *p* < 0.001). Interestingly, the severity of coronary artery stenosis was more obvious in patients with lower HDL-C/apoA-I levels (2.91 ± 0.71 vs. 2.52 ± 0.80 vs. 2.43 ± 0.96, *p* < 0.05).

### 3.5. Effect of the HDL-C/apoA-I Level on MACE Occurrence

**Figure 3:**
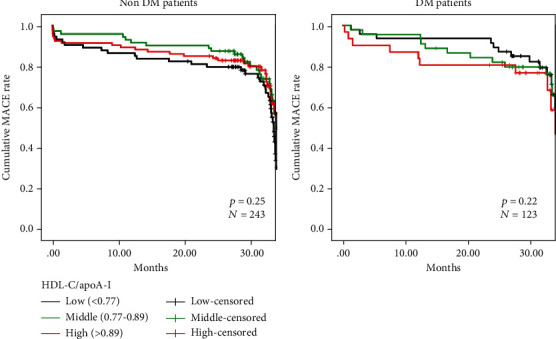
Kaplan-Meier survival curves for freedom from MACE in non-DM and DM patients.

Kaplan-Meier survival analysis was utilized to evaluate the survival curve in different HDL-C/apoA-I level groups in non-DM and DM ACS patients, as shown in Figure 3. However, the analysis showed no difference in MACE in both groups.

## 4. Discussion

The HDL-C/apoA-I ratio has been proposed as a novel surrogate marker for the increased risk of CVD-related, cancer-related, and all-cause death [17]. However, the discriminative value of the HDL-C/apoA-I ratio in predicting the risk and severity of CVD in diabetic and nondiabetic patients has not been studied yet. In this study, we found that the HDL-C/apoA-I ratio was positively correlated with the number of stenotic coronary arteries among diabetic ACS patients but not nondiabetic patients, indicating that the HDL-C/apoA-I ratio may be a valuable marker for predicting the severity of coronary artery stenosis in DM patients. However, the HDL-C/apoA-I ratio exhibited no effect on the 2-year MACE rate in both the diabetic and nondiabetic ACS patients.

The plasma HDL-C level is known to be inversely correlated with the risk of CVD [18, 19]. However, cholesteryl ester transfer protein inhibitors, such as torcetrapib and dalcetrapib, which can increase HDL-C levels by 30-70%, were proven to be ineffective in reducing recurrent cardiovascular events in ACS patients in phase 3 clinical trials [20, 21]. Moreover, increasing evidence demonstrates that not all HDL are functionally equivalent, and HDL dysfunction and remodeling are associated with reduced protective functions [22, 23]. For example, HDL isolated from T2DM and coronary artery disease patients exhibited impaired anti-inflammatory and antioxidative capacities compared to healthy controls [11, 24]. Dysfunctional HDL with elevated proinflammatory but impaired efflux capacities was also associated with an increased incidence of ACS [25]. In the present study, no difference was found in the HDL-C level between the DM and non-DM patients, although the number of stenotic coronary arteries was significantly higher in DM patients. Besides, the HDL-C level was also unchanged in patients with multivessel lesions compared to those with a single-vessel lesion, implying that HDL-C function is likely to be a better target for predicting and decreasing the risk of CVD than HDL-C quantity.

apoA-I is the major protein component of HDL, which plays critical roles in modulating the formation and function of HDL [8]. The HDL-apoA-I exchange (HAE) rate is one of the important approaches to assess the function of HDL, in light of its ability to remodel and release lipid-poor apoA-I [26]. The HAE rate was decreased in T1DM young adults [27] and metabolic syndrome patients [28] compared with healthy control subjects. The HAE rate has also been reported to be inversely associated with the atherosclerotic burden and cardiovascular outcomes in T2DM [29]. Judging from the above studies, it is rational to investigate the value of the HDL-C/apoA-I ratio for cardiovascular outcomes.

Recently, growing evidence has proven that the HDL-C/apoA-I ratio may be an easier approach for estimating HDL function and provide additional insight as a risk marker for CVD. A cross-sectional study of 12,031 men found that the HDL-C level became positively correlated with preclinical atherosclerosis after adjusting for the apoA-I level [30]. The highest HDL-C/apoA-I ratio quartile has been shown to be associated with the increased risk for CVD- and cancer-related deaths [31]. However, a retrospective analysis of 2566 statin-treated coronary artery disease patients reported a controversial result that the increasing level of the HDL-C/apoA-I ratio was associated with less progression of coronary atherosclerosis as evaluated by intravascular ultrasound [32]. In our case, the HDL-C/apoA-I ratio was only increased in DM patients with multivessel lesions and positively correlated with the number of stenotic coronary arteries in DM patients, but not associated with MACEs in diabetic and nondiabetic patients. More well-designed and long-term follow-up studies are still necessary to further investigate the value of HDL-C/apoA-I in the prognosis of ACS patients with and without DM.

## 5. Limitations

Subjects enrolled in this study are limited to patients admitted to the cardiology department of the First Affiliated Hospital of Xi'an Jiaotong University, and the sample size is relatively small. Therefore, the conclusion should be drawn cautiously. A larger cohort study is needed to verify these findings and investigate the role of HDL-C/apoA-I in predicting long-term major adverse cardiac events in diabetic and nondiabetic ACS patients. Moreover, to evaluate the severity of coronary lesions, functional assessment, such as intravascular ultrasound and fractional flow reserve index, should be considered in future studies. Importantly, a complex and systemic score, i.e., SYNTAX score and TIMI score, could be further recorded to predict the severity of coronary artery stenosis more accurately.

## 6. Conclusions

A higher HDL-C/apoA-I ratio is associated with increased severity of coronary artery stenosis in DM patients with ACS. Further studies are needed to clarify the role of the HDL-C/apoA-I ratio in predicting short- and long-term CVD events in these patients.

## Figures and Tables

**Table 1:** Baseline characteristics for non-DM and DM ACS patients.

Characteristics	Whole (*n* = 468)	Non-DM (*n* = 314)	DM (*n* = 154)	*p* value
Age	60.97 ± 9.57	60.59 ± 9.85	61.73 ± 8.94	ns
Male, *n* (%)	365 (78.00)	249 (79.30)	116 (75.32)	ns
BMI (kg/m^2^)	24.95 ± 3.35	24.75 ± 3.35	25.34 ± 3.31	ns
Current smoker, *n* (%)	135 (28.8)	93 (29.62)	42 (27.27)	ns
Family history of CAD, *n* (%)	183 (39.10)	129 (41.08)	54 (35.06)	ns
Hypertension, *n* (%)	255 (54.5)	166 (52.87)	89 (57.79)	ns
Heart rate (bpm)	71.16 ± 16.36	71.10 ± 17.73	71.29 ± 13.20	ns
Systolic BP (mmHg)	126.2 ± 19.03	125.80 ± 18.53	127.02 ± 20.04	ns
Diastolic BP (mmHg)	78.06 ± 11.78	77.59 ± 11.85	79.01 ± 11.62	ns
LVEF (%)	58.27 ± 12.46	58.94 ± 12.36	56.87 ± 12.59	ns
Affected vessels	2.45 ± 0.89	2.39 ± 0.89	2.57 ± 0.87	<0.05
BUN	4.93 ± 1.48	4.84 ± 1.47	5.13 ± 1.50	ns
Creatine (*μ*mol/L)	69.74 ± 20.99	69.10 ± 15.99	71.05 ± 28.61	ns
hsCRP (mg/dL)	2.38 ± 2.33	2.43 ± 2.41	2.28 ± 2.15	ns
HDL-C (mmol/L)	0.91 ± 0.22	0.93 ± 0.23	0.89 ± 0.21	ns
LDL-C (mmol/L)	2.22 ± 0.8	2.26 ± 0.83	2.13 ± 0.74	ns
Triglyceride (mmol/L)	1.68 ± 1.15	1.66 ± 1.21	1.70 ± 1.02	ns
Lipoprotein A (mg/L)	247.74 ± 217.59	255.00 ± 217.61	232.94 ± 217.52	ns
apoA-I (g/L)	1.07 ± 0.19	1.07 ± 0.18	1.08 ± 0.19	ns
HbA1c (%)	6.4 ± 1.31	5.74 ± 0.35	7.75 ± 1.50	<0.001
HDL-C/apoA-I (mmol/g)	0.85 ± 0.12	0.86 ± 0.12	0.82 ± 0.10	<0.01
Medication at discharge				
Aspirin, *n* (%)	462 (98.7)	309 (98.4)	153 (99.4)	ns
Clopidogrel, *n* (%)	448 (95.7)	300 (95.5)	148 (96.1)	ns
Statin, *n* (%)	459 (98.1)	309 (98.4)	150 (97.4)	ns
ACEI/ARB, *n* (%)	417 (89.1)	278 (88.5)	139 (90.3)	ns
Beta-blockers, *n* (%)	410 (87.6)	276 (87.9)	134 (87.0)	ns
CCB, *n* (%)	102 (21.8)	68 (21.7)	34 (22.1)	ns
Main diagnosis				
UA	229 (48.9)	150 (47.77)	79 (51.30)	ns
NSTEMI	71 (15.2)	50 (15.92)	21 (13.64)	ns
STEMI	168 (35.9)	114 (36.31)	54 (35.06)	ns

Data are mean ± SD and number (%). ACS: acute coronary syndrome; ACEI: angiotensin-converting enzyme inhibitor; apoA-I: apolipoprotein A-I; ARB: angiotensin receptor blocker; BMI: body mass index; BP: blood pressure; BUN: blood urea nitrogen; CAD: coronary artery disease; CCB: calcium channel blocker; CKMB: creatine kinase MB; DM: diabetes mellitus; HbA1c: hemoglobin A1c; HDL-C: high-density lipoprotein cholesterol; hsCRP: high-sensitivity C-reactive protein; LDL-C: low-density lipoprotein cholesterol; LVEF: left ventricular ejection fraction; NSTEMI: non-ST elevated myocardial infarction; STEMI: ST elevated myocardial infarction; UA: unstable angina.

**Table 2:** Baseline characteristics for different ACS patients with single-vessel (1-2 affected vessels) or multivessel (≥3 affected vessels) lesions.

Characteristics	Affected vessels		*p* value
	1–2	≥3	
All			
Patient number, *n* (%)	213 (45.5)	255 (54.5)	
Age	60.28 ± 9.36	61.22 ± 9.65	ns
Male, *n* (%)	154 (75.9)	196 (80.7)	ns
BMI (kg/m^2^)	24.94 ± 3.43	24.94 ± 3.28	ns
Heart rate (bpm)	70.56 ± 13.74	72.01 ± 18.62	ns
Systolic BP (mmHg)	124.81 ± 17.11	126.76 ± 19.86	ns
Diastolic BP (mmHg)	77.75 ± 11.01	78.35 ± 12.38	ns
LVEF (%)	59.65 ± 11.46	57.26 ± 13.18	ns
Creatine (*μ*mol/L)	69.42 ± 24.51	70.3 ± 17.91	ns
HDL-C (mmol/L)	0.92 ± 0.22	0.9 ± 0.21	ns
LDL-C (mmol/L)	2.18 ± 0.8	2.24 ± 0.77	ns
Triglyceride (mmol/L)	1.69 ± 1.15	1.66 ± 1.14	ns
Lipoprotein A (mg/L)	224.96 ± 191.36	265.6 ± 233.81	ns
apoA-I (g/L)	1.09 ± 0.17	1.05 ± 0.18	<0.05
HbA1c (%)	6.24 ± 1.14	6.57 ± 1.45	<0.05
HDL-C/apoA-I (mmol/g)	0.84 ± 0.11	0.85 ± 0.11	ns
Non-DM			
Patient number, *n* (%)	149 (47.5)	165 (52.5)	
Age	59.3 ± 9.71	61.2 ± 9.85	ns
Male, *n* (%)	107 (77.0)	130 (83.3)	ns
BMI (kg/m^2^)	24.71 ± 3.37	24.77 ± 3.34	ns
HR (bpm)	70.69 ± 13.88	71.85 ± 21.14	ns
Systolic BP (mmHg)	124.36 ± 16.1	126.03 ± 19.44	ns
Diastolic BP (mmHg)	77.21 ± 11.21	77.9 ± 12.4	ns
LVEF (%)	60.34 ± 11.13	57.8 ± 13.49	ns
Creatine (*μ*mol/L)	67.56 ± 15.56	70.84 ± 15.96	ns
HDL-C (mmol/L)	0.95 ± 0.22	0.9 ± 0.21	ns
LDL-C (mmol/L)	2.18 ± 0.84	2.31 ± 0.77	ns
Triglyceride (mmol/L)	1.69 ± 1.28	1.62 ± 1.12	ns
Lipoprotein A (mg/L)	231.18 ± 198.69	277.15 ± 226.46	ns
apoA-I (g/L)	1.1 ± 0.17	1.05 ± 0.18	<0.05
HbA1c (%)	5.7 ± 0.34	5.76 ± 0.35	ns
HDL-C/apoA-I (mmol/g)	0.86 ± 0.12	0.86 ± 0.1	ns
DM			
Patient number, *n* (%)	64 (41.6)	90 (58.4)	
Age	62.39 ± 8.24	61.25 ± 9.33	ns
Male, *n* (%)	47 (73.4)	66 (75.9)	ns
BMI (kg/m^2^)	25.52 ± 3.55	25.22 ± 3.18	ns
HR (bpm)	70.28 ± 13.54	72.29 ± 13.06	ns
Systolic BP (mmHg)	125.76 ± 19.21	128.06 ± 20.65	ns
Diastolic BP (mmHg)	78.92 ± 10.54	79.16 ± 12.37	ns
LVEF (%)	58.19 ± 12.08	56.25 ± 12.59	ns
Creatine (*μ*mol/L)	73.44 ± 37.03	69.33 ± 21.03	ns
HDL-C (mmol/L)	0.87 ± 0.21	0.9 ± 0.2	ns
LDL-C (mmol/L)	2.18 ± 0.73	2.11 ± 0.75	ns
Triglyceride (mmol/L)	1.69 ± 0.76	1.72 ± 1.18	ns
Lipoprotein A (mg/L)	211.45 ± 175.11	244.89 ± 246.42	ns
apoA-I (g/L)	1.08 ± 0.18	1.07 ± 0.18	ns
HbA1c (%)	7.41 ± 1.38	8.03 ± 1.54	<0.05
HDL-C/apoA-I (mmol/g)	0.79 ± 0.08	0.84 ± 0.11	<0.05

Data are mean ± SD and number (%). ACS: acute coronary syndrome; apoA-I: apolipoprotein A-I; BMI: body mass index; BP: blood pressure; DM: diabetes mellitus; HbA1c: hemoglobin A1c; HDL-C: high-density lipoprotein cholesterol; LDL-C: low-density lipoprotein cholesterol; LVEF: left ventricular ejection fraction.

**Table 3:** Multiregression analysis of the severity of coronary artery stenosis in non-DM and DM ACS patients.

Variable	Coefficient	95% CI	*p* value
Non-DM			
Age	0.012	-0.002 to 0.025	ns
BMI (kg/m^2^)	0.006	-0.033 to 0.045	ns
HDL-C/apoA-I (mmol/g)	-0.091	-1.263 to 1.080	ns
apoA-I (g/L)	-0.299	-0.986 to 0.387	ns
HbA1c (%)	0.178	-0.166 to 0.521	ns
DM			
Age	-0.019	-0.038 to 0.000	ns
BMI (kg/m^2^)	0.005	-0.044 to 0.054	ns
HDL-C/apoA-I (mmol/g)	1.801	0.235 to 3.368	<0.05
apoA-I (g/L)	-0.317	-1.252 to 0.618	ns
HbA1c (%)	0.052	-0.057 to 0.162	ns

Data are the mean ± SD and number (%). apoA-I: apolipoprotein A-I; BMI: body mass index; BP: blood pressure; DM: diabetes mellitus; HbA1c: hemoglobin A1c; HDL-C: high-density lipoprotein cholesterol.

**Table 4:** Baseline characteristics for ACS patients with or without DM in HDL/apoA tertiles.

Characteristics	HDL/apoA			*p* value
	<0.77	0.77-0.89	>0.89	
All				
Patient number	152	161	155	
Age	58.57 ± 8.49	60.64 ± 9.95	63.65 ± 9.52	<0.001
Male, *n* (%)	121 (79.6)	125 (77.6)	119 (76.8)	ns
BMI (kg/m^2^)	25.78 ± 3.42^∗^	24.93 ± 3.14	24.05 ± 3.27	<0.01
HR (bpm)	69.98 ± 19.05	70.95 ± 12.87	72.52 ± 16.74	ns
Systolic BP (mmHg)	125 ± 18.58	127.33 ± 17.88	126.2 ± 20.58	ns
Diastolic BP (mmHg)	78.3 ± 11.39	78.83 ± 11.16	77 ± 12.73	ns
LVEF (%)	59.06 ± 12.57	58.83 ± 11.74	56.93 ± 13	ns
Creatine (*μ*mol/L)	69.2 ± 16.94	70.69 ± 26.76	69.27 ± 17.56	ns
HDL-C (mmol/L)	0.75 ± 0.11	0.9 ± 0.15	1.08 ± 0.23	<0.001
LDL-C (mmol/L)	2.03 ± 0.62	2.26 ± 0.78	2.35 ± 0.93	<0.01
Triglyceride (mmol/L)	2.34 ± 1.48	1.54 ± 0.72	1.15 ± 0.73	<0.001
Lipoprotein A (mg/L)	197.66 ± 192.03	251.75 ± 204.74	292.67 ± 243.59	<0.05
apoA-I (g/L)	1.03 ± 0.14	1.07 ± 0.17	1.11 ± 0.22	<0.01
HbA1c (%)	6.45 ± 1.27	6.52 ± 1.52	6.22 ± 1.06	<0.01
HDL-C/apoA-I (mmol/g)	0.73 ± 0.05^∗^	0.83 ± 0.02^∗^	0.97 ± 0.08^∗^	<0.001
Affected vessels	2.38 ± 0.94	2.43 ± 0.84	2.54 ± 0.88	ns
Non-DM				
Patient number	92	103	110	
Age	58.35 ± 8.68	59.26 ± 10.21	63.48 ± 9.76	<0.001
Male, *n* (%)	75 (81.5)	84 (81.6)	90 (75.6)	ns
BMI (kg/m^2^)	25.93 ± 3.18	24.74 ± 3.09	23.73 ± 3.42	<0.001
HR (bpm)	70.08 ± 22.96	70.45 ± 12.74	72.46 ± 16.86	ns
Systolic BP (mmHg)	124.97 ± 17.14	125.95 ± 16.35	126.3 ± 21.26	ns
Diastolic BP (mmHg)	78.58 ± 11.72	78.24 ± 11.03	76.27 ± 12.58	ns
LVEF (%)	59.55 ± 12.56	60.12 ± 11.11	57.5 ± 13.14	ns
Creatine (*μ*mol/L)	69.59 ± 15.69	68.81 ± 15.04	68.96 ± 17.1	ns
HDL-C (mmol/L)	0.75 ± 0.13	0.9 ± 0.14	1.08 ± 0.24	<0.001
LDL-C (mmol/L)	2.09 ± 0.67	2.3 ± 0.81	2.37 ± 0.94	ns
Triglyceride (mmol/L)	2.44 ± 1.7	1.53 ± 0.57	1.18 ± 0.81	<0.001
Lipoprotein A (mg/L)	194.42 ± 162.65	251.64 ± 204.09	304.74 ± 252.65	<0.01
apoA-I (g/L)	1.03 ± 0.15	1.07 ± 0.16	1.11 ± 0.22	<0.05
HbA1c (%)	5.75 ± 0.37	5.72 ± 0.38	5.76 ± 0.32	ns
HDL-C/apoA-I (mmol/g)	0.73 ± 0.05	0.84 ± 0.03	0.98 ± 0.09	<0.001
Affected vessels	2.34 ± 0.93	2.38 ± 0.86	2.43 ± 0.90	ns
DM				
Patient number	60	58	36	
Age	58.92 ± 8.27	63.1 ± 9.05	64.22 ± 8.79	<0.05
Male, *n* (%)	46 (76.7)	41 (70.7)	29 (80.6)	ns
BMI (kg/m^2^)	25.54 ± 3.8	25.26 ± 3.24	25.1 ± 2.55	ns
HR (bpm)	69.85 ± 10.8	71.86 ± 13.17	72.75 ± 16.61	ns
Systolic BP (mmHg)	125.05 ± 20.77	129.76 ± 20.25	125.89 ± 18.44	ns
Diastolic BP (mmHg)	77.9 ± 10.98	79.9 ± 11.41	79.44 ± 13.09	ns
LVEF (%)	58.3 ± 12.68	56.53 ± 12.6	55.09 ± 12.54	ns
Creatine (*μ*mol/L)	68.6 ± 18.83	74.03 ± 39.85	70.33 ± 19.22	ns
HDL-C (mmol/L)	0.76 ± 0.11	0.9 ± 0.17	1.08 ± 0.24	<0.001
LDL-C (mmol/L)	1.96 ± 0.55	2.21 ± 0.75	2.3 ± 0.94	ns
Triglyceride (mmol/L)	2.21 ± 1.1	1.57 ± 0.94	1.07 ± 0.41	<0.001
Lipoprotein A (mg/L)	202.64 ± 231.38	251.94 ± 207.69	252.82 ± 209.19	ns
apoA-I (g/L)	1.05 ± 0.12	1.08 ± 0.19	1.12 ± 0.25	ns
HbA1c (%)	7.54 ± 1.41	7.97 ± 1.72	7.76 ± 1.22	ns
HDL-C/apoA-I (mmol/g)	0.73 ± 0.05	0.83 ± 0.03	0.97 ± 0.07	<0.05
Affected vessels	2.43 ± 0.96	2.52 ± 0.80	2.91 ± 0.71	<0.05

Data are mean ± SD and number (%). ACS: acute coronary syndrome; apoA-I: apolipoprotein A-I; BMI: body mass index; BP: blood pressure; DM: diabetes mellitus; HbA1c: hemoglobin A1c; HDL-C: high-density lipoprotein cholesterol; LDL-C: low-density lipoprotein cholesterol; LVEF: left ventricular ejection fraction.

## Data Availability

Data supporting the conclusions of this article are included within the article and available from the corresponding authors on reasonable request.

## Abbreviations

ACS: Acute coronary syndrome
apoA-I: Apolipoprotein A-I
BP: Blood pressure
BUN: Blood urea nitrogen
HbA1c: Hemoglobin A1c
HDL-C: High-density lipoprotein cholesteryl
HR: Heart rate
LDL-C: Low-density lipoprotein cholesteryl
LVEF: Left ventricular ejection fraction
MACE: Major adverse cardiac event
NSTEMI: Non-ST elevated myocardial infarction
STEMI: ST elevated myocardial infarction
T2DM: Type 2 diabetes mellitus
UA: Unstable angina.
